# Case-Only Designs in Pharmacoepidemiology: A Systematic Review

**DOI:** 10.1371/journal.pone.0049444

**Published:** 2012-11-16

**Authors:** Sandra Nordmann, Lucie Biard, Philippe Ravaud, Marina Esposito-Farèse, Florence Tubach

**Affiliations:** 1 Univ Paris Diderot, Sorbonne Paris Cité, UMR-S 738; INSERM, UMR-S 738, Paris, France; 2 APHP, Hôpital Bichat, Département d’Epidémiologie et Recherche Clinique, Paris, France; 3 INSERM, UMR-S 738; APHP, Hôpital Hôtel-Dieu, Centre d’Epidémiologie Clinique; Université Paris Descartes, Paris, France; 4 INSERM, CIE 801; APHP, Hôpital Bichat, Département d’Epidémiologie et Recherche Clinique, Paris, France; 5 Univ Paris Diderot, Sorbonne Paris Cité; INSERM, UMR-S 738; INSERM, CIE 801; APHP, Hôpital Bichat, Département d Epidémiologie et Recherche Clinique, Paris, France; University of Ottawa, Canada

## Abstract

**Background:**

Case-only designs have been used since late 1980’s. In these, as opposed to case-control or cohort studies for instance, only cases are required and are self-controlled, eliminating selection biases and confounding related to control subjects, and time-invariant characteristics. The objectives of this systematic review were to analyze how the two main case-only designs – case-crossover (CC) and self-controlled case series (SCCS) – have been applied and reported in pharmacoepidemiology literature, in terms of applicability assumptions and specificities of these designs.

**Methodology/Principal Findings:**

We systematically selected all reports in this field involving case-only designs from MEDLINE and EMBASE up to September 15, 2010. Data were extracted using a standardized form. The analysis included 93 reports 50 (54%) of CC and 45 (48%) SCCS, 2 reports combined both designs. In 12 (24%) CC and 18 (40%) SCCS articles, all applicable validity assumptions of the designs were fulfilled, respectively. Fifty (54%) articles (15 CC (30%) and 35 (78%) SCCS) adequately addressed the specificities of the case-only analyses in the way they reported results.

**Conclusions/Significance:**

Our systematic review underlines that implementation of CC and SCCS designs needs to be more rigorous with regard to validity assumptions, as well as improvement in results reporting.

## Introduction

Because of the continued increase in the use of therapeutic drugs, the development of accurate and efficient methods is critical to study potential adverse effects. Cohort and case-control studies are widely accepted designs for the evaluation of the risks and benefits of post-licensure medications. However, these designs are vulnerable to confounding and selection bias. In the late 1980’s, alternative methods relying only on cases (i.e. without controls), termed case-only designs, were introduced to attempt to avoid some of these limitations. Case-only designs are attractive because the cases are self-matched, which eliminates time-invariant confounders. They are generally less expensive, shorter in time, and simpler to carry out than conventional designs.

Among existing case-only designs, 5 have been used in pharmacoepidemiology: the case-crossover design (CC) [1], the case-time-control design (CTC) [2], the self-controlled case series design (SCCS), originally called case series analysis [3], the screening method [4] and the prescription sequence symmetry analysis (PSSA) also called the symmetry principle [5] (See Appendix S1, for a description of the designs). Of those designs, CC and SCCS have been used the most.

The CC design was introduced by Maclure in 1991 to study the short-term effects of intermittent exposures on the risk of acute events [1]. A risk period is defined as a time period preceding the event of interest (such as gastro-intestinal bleeding for instance). If the patient is exposed during this period, the exposure (medicine treatment) will be considered to be related to the event. In most cases, the risk period immediately precedes the event. On the other hand, control periods are defined before or after the event as time periods during which exposure to the drug of interest is not related to the event. Due to physiological reasons, control periods are generally chosen remote in time from the event. With this design, the probability of exposure in the risk period is compared to the probability of exposure in control period(s). Since long-term exposures could lead to bias [6]–[7], particularly in the case of time-varying exposure [8], Suissa extended the CC with the CTC design in 1995 [2]. In the CTC design, the CC odds ratio has been adjusted to the time-trend of the exposure, which is measured with an independent control group. Farrington proposed the SCCS design in 1995 [3] to assess post-licensure adverse events related to vaccines, and more generally associations between acute outcomes and transient exposures [9]. Here, risk periods are the periods during and/or after each occurrence of exposure, in which people are considered to be at greater risk of the event, whereas control periods include all other time periods of the observation period, in which people are considered to be at baseline risk. The incidences of events within risk periods are compared to those within control periods, allowing for age or time effects.

CC and SCCS designs are considered suitable tools in post-licensure pharmacoepidemiological studies. With the development of health information technology and the use of large healthcare databases to retrieve real-life data of exposure/event occurrence, these designs seem particularly appropriate to analyze pharmacovigilance data. However, medication use patterns may not correspond to the exposure characteristics for which these designs were developed. For instance, a medication may be a chronic rather than transient exposure. Also, adverse events may be permanent or generate chronic consequences that might influence further exposure [10]. Applicability of the design to specific settings is a key issue to be addressed by investigators and authors. Furthermore, case-only statistical methods have specificities and differ from conventional ones such as case-control or cohort analyses (See Appendix S1). Reporting has to present adequate specific information for the consistency of the study results to be assessed.

We performed a systematic review of the use and reporting of case-only designs in the pharmacoepidemiology literature, focusing on design applicability and reporting, in studies involving the two most widely used case-only designs, CC (including its extension CTC) and SCCS.

## Methods

### Search Strategy

We identified reports of pharmacoepidemiological studies involving a case-only design published up to September 15, 2010, in English, in MEDLINE via PubMed and EMBASE databases. The search keywords were “case-crossover” OR “case-time-control” OR “self-controlled case series” OR “case series analysis” OR (“self control” AND “case series”) OR “prescription sequence symmetry analysis” OR (“sequence symmetry” AND “analysis”) OR (“case coverage” AND “screening method”). At the time of the search, since the term “case-only” was mainly used in gene-environment interaction studies, it was not added as a keyword [11]–[12]. Additional articles were sought from the reference lists of selected articles and from the SCCS method website [13]. This website provides methodology information and direct links to articles related to the SCCS design, as well as statistical software instructions for implementation of SCCS analyses. The site has been developed by H.Whitaker, a member of the team of P.Farrington at the Department of Statistics at the Open University, England.

No formal protocol was specified.

### Eligibility Criteria and Screening Process

Two of the authors (SN and LB) screened titles and abstracts to identify potentially relevant articles. A final selection was made after reading the full texts. Articles were included if CC, CTC or SCCS case-only designs were used and if the purpose of the study was pharmacoepidemiology, i.e. the objective was to assess either human safety or efficacy of drugs, or medical devices. Reviews, meta-analyses, letters to the editor, reports of randomized trials, articles reporting methodological issues only and reports of cost-effectiveness studies were not included. In the case of duplicate publication (the same study using the same design described in several articles), only the article that was published first was selected.

### Data Collection

We created a standardized data extraction form (Appendix S2), with items selected by analysis of the literature on case-only designs, and of the “Strengthening the Reporting of Observational Studies in Epidemiology” (STROBE) guidelines for reporting observational epidemiological studies [14].

Before full data extraction, three independent reviewers (SN, LB and FT) performed a calibration exercise for the extraction form on a random set of ten reports. Any disagreements were resolved by consensus. Then, two independent reviewers (SN and LB) completed data extraction. Journal and authors’ names were not concealed to reviewers.

The data extraction form covered the following fields: ‘general characteristics’, ‘methods’ and ‘results’ according to the standard sections of reports (for details, see Appendix S2: Data Extraction Form).

The ‘general characteristics’ section of the form included the year of publication, study design (number and type of designs used) and rationale for using a case-only design. Since CTC design is an extension of CC, reports using a CTC design were included as using CC in the analysis.

The ‘methods’ section contained several subsections. First, we collected data regarding validity assumptions for the use of a case-only analysis. To that aim, we consulted 3 CC and three SCCS experts to identify validity assumptions for each design. The experts had published at least two articles reporting studies involving the case-only design of interest (i.e., CC or SCCS), and two articles related to methodological issues of that case-only design. Two of the experts were the initial developers of CC and SCCS designs. First, experts were consulted on a list of suggested validity assumptions that one of the investigators (SN) had selected from the literature. They were asked whether they agreed with them as major validity assumptions for the corresponding design and we requested additions, deletions or alternate wording if necessary. In a second round, we submitted the resulting criteria to the same panel of experts. The final list of assumptions for the CC design was as follows: acute event, rare event, intermittent exposure, same probability of event occurrence during case and control periods, no time-trend in exposure (for instance, a time-trend would be seasonality in exposure or change in prescription habits [10], [15]–[16], whereas the underlying probability of exposure should be constant over time intervals to consider a CC analysis) or use of a CTC in the case of time-trend. The final list of assumptions for the SCCS design was as follows: rare or recurrent event, independence between two consecutive events (if event is recurrent), intermittent exposure, probability of further exposure not affected by previous events, probability of short-term mortality not affected by events (to ensure that control periods censoring is not event-dependent), events and exposures data collected independently. An event was considered rare on the basis of the population studied: events related to chronic disease progression were not considered rare if the patients included all had the disease; events were considered rare if the study sample was selected from the general population.

Secondly, the ‘methods’ section of the data extraction form included the type of exposure (vaccines, other medication and medical device were the main categories). Sources of exposure and event data were also collected (administrative database, data collected specifically for the study and data from pre-existing studies were the main categories). The next ‘methods’ subsection consisted of the characteristics of the risk period, including: the rationale for its definition (a rationale was considered valid if it was based on physiological evidence or on reference(s) to previous works or validated by experts), number of risk periods if appropriate, duration, identical period for all subjects or not, and time restriction(s) within the period. Characteristics of the control periods were collected in the same manner.

The last part of the ‘methods’ section contained statistical considerations, including: the sample size calculation (focusing on pharmacoepidemiological studies, we awaited sample size calculation to be addressed), model and risk estimate, and sensitivity analyses. For CC, the adequate model is conditional logistic regression and adequate risk estimators are odds ratio (OR), or rate ratio (RR) (if the event was rare), incidence rate ratio (if rate ratios were available) and relative risk. For SCCS, the adequate model is a conditional Poisson regression and adequate risk estimators are relative risk, relative incidence, rate ratio and incidence rate ratio.

The ‘results’ section of the form focused on whether sufficient information was provided with regard to design specificities. For CC designs, in addition to the OR/RR and its 95% confidence interval, adequate information is the number of subjects with discordant periods (unexposed control period with exposed risk period and vice versa), i.e. the count of subjects relevant to the analysis. For SCCS designs, adequate information is the count of events in risk and control periods, separately. For articles presenting results from several designs on the same data, statistical conclusions across analyses were also collected. Statistical conclusions were considered as similar if both designs failed to reject the null or both rejected the null in the same direction.

### Statistical Analysis

Statistical analysis was performed with R software v2.12.0 (R Development Core Team, R Foundation for Statistical Computing). As for descriptive analysis, categorical variables were described with frequencies and percentages. Fisher’s exact test was performed, to compare categorical variables at a 5% significance level for exploratory analyses, when appropriate.

## Results

**Figure 1:**
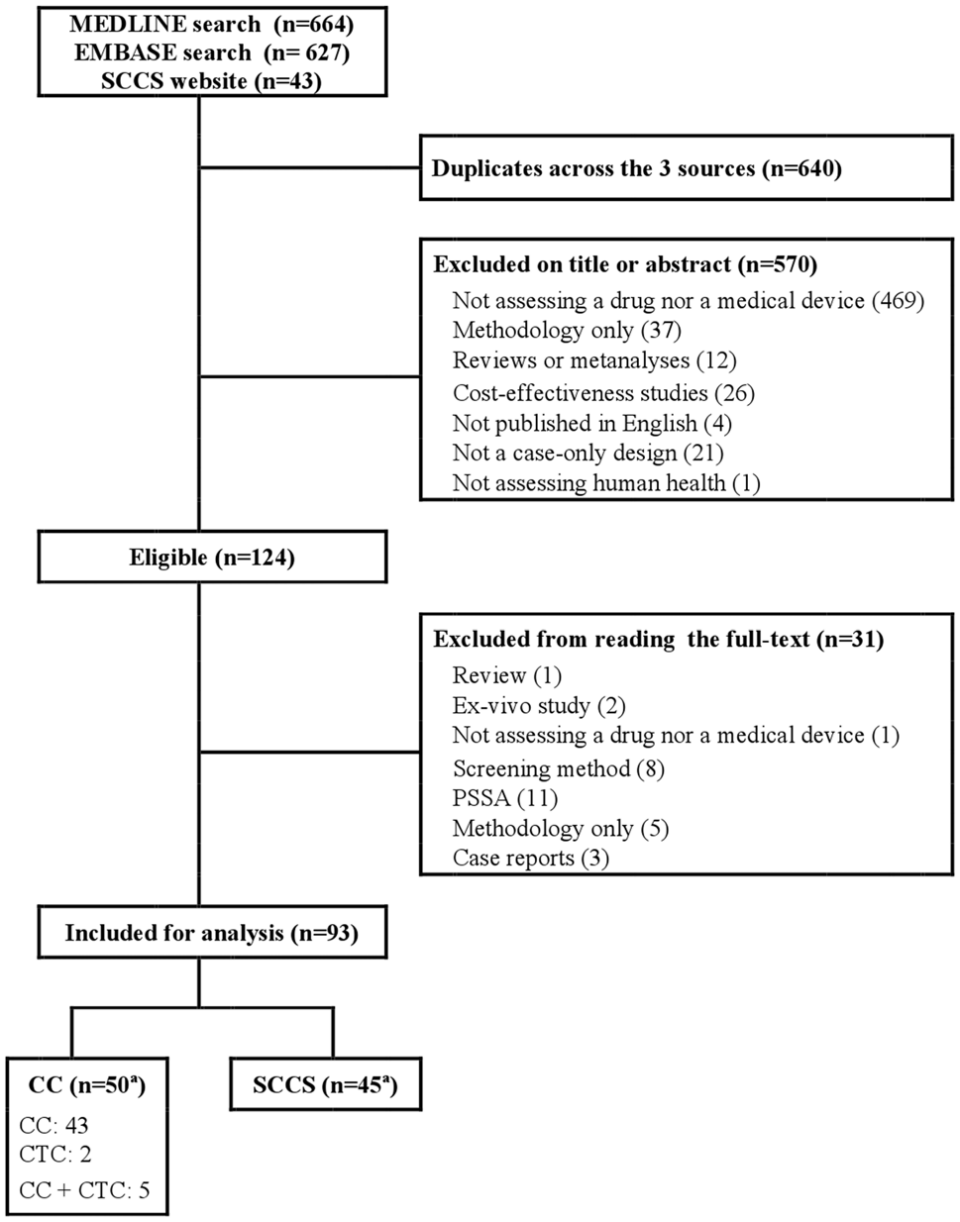
Flowchart of selected articles.

A flowchart of the selection of articles is included in Figure 1. A search of MEDLINE yielded 664 citations and 627 were found in EMBASE. In addition, 43 reports were identified on the SCCS method website. After excluding 640 duplicates, 694 articles remained from the 3 sources, among which 164 reports were considered eligible, based on titles and abstracts. Finally, 93 reports were included for analysis after reading the full texts: 50 articles reporting studies using CC or CTC (7 studies used CTC designs) and 45 using SCCS. Two articles reported both CC and SCCS designs. (See Appendix S3 for a list of complete references for the reports included).

**Figure 2:**
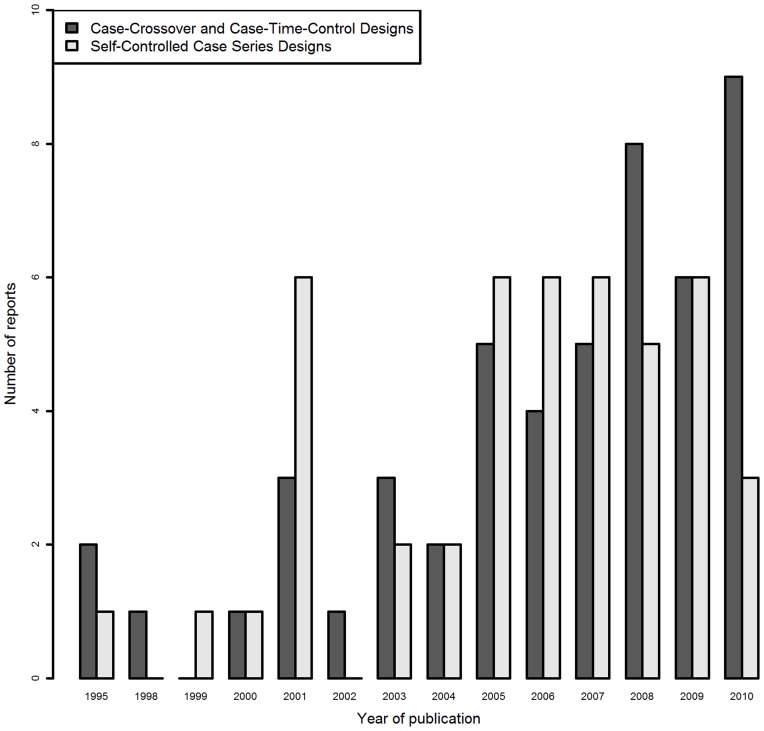
Number of reports per year of studies using case-crossover or self-controlled case series designs.

Regarding the general characteristics of the studies included (Table 1), the first article using a case-only design in pharmacoepidemiology was published in 1995. The annual number of published articles reporting case-only studies increased over time until 2005. Since then, SCCS design use has remained stable, but CC use has continued to rise (Figure 2). In 39 reports (42%), several designs were combined; 4 reports involved 3 designs (CC, CTC and case-control) and 2 reported combined CC and SCCS designs. The rationale for using a case-only design was reported in 75 articles (81%). In 55%, the purpose was to limit bias (confounding or selection biases).

**Table 1:** General Characteristics of the Studies Using Case-Crossover and/or Self-Controlled Case Series Designs.

	Case-Crossover(n = 50)	Self-Controlled Case Series(n = 45)	All Articles(n = 93)^a^
	*No. (%)*	*No. (%)*	*No. (%)*
Designs	50 (100)	45 (100)	93 (100)^a^
Several designs were used	24 (48)	17 (38)	39 (42) a
Additional case-only study	2 SCCS	2 CC	2 (2) a
Additional case-control study	13 (26)	6 (13)	19 (20)
Additional cohort study	7 (14)	5 (11)	12 (13)
Additional case-time-control study coupled to CC design	5 (10)		5 (5)
Additional ecological study	2 (4)	3 (7)	5 (5)
Rationale for the use of a case-only design	43 (86)	34 (76)	75 (81) a
To limit bias^b^	30 (60)	23 (51)	51 (55) a
Practical issues^c^	21 (42)	19 (42)	38 (41) a
Justification related to event or exposure characteristics	25 (50)	8 (18)	33 (35)

a Two reports used both CC and SCCS.

b e.g.: bias due to fixed-confounders.

c e.g.: suitable database, no representative control group available, necessity of easy, rapid, simple design.

As for the methodological characteristics of the studies, 12 CC studies (24%) fulfilled all 5 validity assumptions for this design (Table 2). Twenty-one studies (42%) fulfilled 4 assumptions, 14 studies (28%) 3 assumptions and 3 studies (6%) only 2 assumptions. Overall, 33 CC studies (66%) fulfilled at least 4 of the 5 validity assumptions. Eighteen SCCS articles (40%) fulfilled all 6 assumptions, 15 (33%) fulfilled all but one, 6 (13%) only 4 assumptions, and 6 (13%) ≤3 assumptions. In all, 33 SCCS articles (73%) fulfilled at least 5 of the 6 assumptions. In reports involving case-only analyses and other designs, the proportion of reports with similar statistical conclusions across designs did not statistically differ between reports where all assumptions for the case-only analysis were fulfilled (n = 9, 60%) from the others (n = 17, 65%; p = 0.95).

**Table 2:** Validity Assumptions for Use of Case-Crossover and Self-Controlled Case Series Designs.

Case-crossover studies	N = 50
Assumptions	Studies *No.(%)*
Acute outcome	43 (86)
Rare event	32 (64)
Intermittent exposure	36 (72)
The opportunity of event is the same during case and control time-period(s)	47 (94)
No time-trend in exposure or CTC use	34 (68)
All assumptions fulfilled	12 (24)
**Self-controlled case series**	**N = 45**
**Assumptions**	**Studies ** ***No. (%)***
Rare or recurrent event	45 (100)
Only one event per subject or independence between two consecutive events was defined	39 (87)
Intermittent exposures	35 (78)
Probability of further exposure not affected by previous events	24 (53)
Event did not affect the short term mortality probability	39 (87)
Collections of events and exposures was independent	42 (93)
All assumptions fulfilled	18 (40)

The exposures were predominantly vaccines in SCCS reports and medications in CC reports (Table 3). Exposure and event data were most frequently collected from administrative databases. Some studies combined several data sources, either for exposure or event. For CC studies, 18 articles (36%) gave a valid rationale for the definition of the risk period and 23 articles (51%) for SCCS studies (Table 4). The number of control periods was clearly reported in 41 CC articles (82%). Time restrictions were introduced in the risk periods in 5 articles, and in the control periods in 3 of them (same restrictions).

**Table 3:** Characteristics of Exposures and Data Sources of the Studies.

		Case-Crossover(n = 50)	Self-controlled case series(n = 45)	All Articles(n = 93)^a^
		*No.(%)*	*No.(%)*	*No.(%)*
Exposures				
Vaccines		3 (6)	34 (76)	36 (39)^a^
Other medication		44 (88)	11 (24)	54 (58)
Medical device (gloves, condom)		3 (6)	0	3 (3)
Data sources				
Sources of exposure data				
Administrative database b		37 (74)	34 (76)	69 (74)^a^
Data collected for the study c		13 (26)	4 (9)	17 (19)
Pre-existing studies^d^		6 (12)	3 (7)	9 (10)
Sources of events data collection				
Administrative database b		39 (78)	41 (91)	78 (84)^a^
Data collected for the study c		3 (6)	1 (2)	4 (4)
Pre-existing studies^d^		12 (24)	4 (9)	16 (17)
Events and exposures data resulted from administrative databases		33 (66)	33 (73)	64 (69)^a^

a Two reports used both CC and SCCS.

b Administrative database: reimbursement database, hospital or institutional records, primary care database (THIN, GRPD).

c Data collected for the study: self-questionnaire, diary, telephone call, web site, interview, individual health booklet.

d Pre-existing studies: register, clinical/cohort data.

**Table 4:** Characteristics of Risk and Control Periods.

	Case-Crossover(n = 50)	Self-controlled caseseries (n = 45)
	*No.(%)*	*No.(%)*
Rationale for definition of the risk period		
Based on practical issues	8 (16)	4 (9)
A valid rationale was given for the definition (at least physiological evidences or reference/expert)	18 (36)	23 (51)
Risk period characteristics		
Each subject had the same risk period duration (for CC)	41 (82)	
Range duration of the risk period in days (calculated in 44 CC and 39 SCCS articles respectively)	0,1–364	4–1770
Control period characteristics (for CC)		
Number of control period(s) was reported	41 (82)	
Range duration of the control period(s) in days	0–365	
Each subject had the same control period(s) duration	38 (76)	
Control period(s) selection		
Only before the risk period	34 (68)	
Before or after the risk period	5 (10)	
Others	5 (10)	
All the observation period but the risk period	5 (10)	35 (78)
Unclear	1 (2)	3 (7)
Pre-specified period		7 (16)
Risk period(s) and control period(s) had the same time restrictions	3/5	

Concerning statistical issues, a sample size calculation [17], [18] was reported in 10 articles (11%) (Table 5). Administrative databases usually provide large sample sizes and may be considered somewhat satisfying with regard to power considerations. Also, when a study presents a case-only design as exploratory analysis, the main design (cohort, case-control) may have provided a sample size calculation. Among the 54 studies that did not combine a case-only design with other(s), only 2 (6%) reported a sample size calculation. In this review, 6 articles (6%) did not correspond to either of those situations (administrative database or multiple designs). None of them reported a sample size calculation. Forty-one (82%) CC and 23 (51%) SCCS articles reported using the adequate statistical model [1], [18], [19] (Table 5). Overall, the statistical model was unclear in almost one-third of the articles. However, most articles reported the appropriate risk estimator: CC, 48 (96%); and SCCS, 44 (98%). Sensitivity analyses were reported in 37 articles (40%).

**Table 5:** Statistical Issues.

	Case- Crossover(n = 50)	Self-Controlled Case Series(n = 45)	All Articles(n = 93^a^)
	*No.(%)*	*No.(%)*	*No.(%)*
Sample size calculation reported	5 (10)	6 (13)	10 (11)
Statistical model			
Adequate model b	41 (82)	23 (51)	62 (67)^a^
Model unclearly reported	7 (14)	22 (50)	29 (31)
Statistical model not relevant	2 (4)		2 (2)
Reported estimator of the strength of the association			
Adequate risk estimator^c^	48 (96)	44 (98)	90 (97)^a^
Risk estimator not adequate or unclear	2 (4)	1 (2)	3 (3)
Sensitivity analyses reported	22 (44)	16 (36)	37 (40)^a^
Risk period duration variation	9 (18)	7 (16)	16 (17)
Control period characteristics (number, duration or onset) variation	9 (18)	7 (16)	15 (16) a
Exposures and/or events	12 (24)	7 (16)	19 (20)
Person-times number or duration variation	2 (4)	0	2 (2)

a One report used both CC and SCCS.

b Conditional logistic regression for the CC and conditional Poisson regression for SCCS.

c CC: Odds Ratio, Relative risk, Rate Ratio/Incidence Rate Ratio; SCCS: Relative Risk, Relative Incidence, Incidence Rate Ratio.

Regarding ‘results’ sections of the included articles, 50 (54%) articles (15 CC (30%) and 35 (78%) SCCS) reported the adequate information to account for their being case-only analyses (Table 6). Sixteen (17%) reports presented insufficient information, that is the risk estimate with its confidence interval only (10 (20%) CC and 6 (13%) SCCS). Twenty-seven (29%) provided inadequate details: 24 (48%) CC designs reported the count of exposed and non-exposed risk periods and 3 (7%) SCCS designs reported the count of events, irrespective of risk/control periods.

**Table 6:** Main Specific Methodological Points to Consider in Planning and Reporting Case-only Studies (to be considered as a complement of the STROBE Statement).

Points to Consider	Recommendation for Planning	Recommendation for Reporting
1. Applicability of case-only designto the study objective (eventand exposure studied)	Study should be planned in order to fulfil the validity assumptions of the design.	Report that setting is valid for the design implementation.In case of unfulfilled assumptions: lack of fulfilment should be stated, impact on results should be discussed, and if possible, comparison with other design(s) performed.
2. Risk and controlperiods’ definition	Risk and control periods definition have to be based onphysiological evidence or hypotheses, referenced orvalidated by an expert group^12^. Risk period should beidentical for all subjects. Sensitivity analysis on risk/controlperiods onset, end and duration should be planned.	Report the risk period definition and the justification of their characteristics. Report sensitivity analyses results varying risk/control periods’ characteristics.
	Restriction times should be implemented if appropriate.They should be identical in risk and control periods	Report rationale for introducing restriction times if any.
3. Statistical issues		
Statistical model	Conditional logistic regression for CC and Poissonregression for SCCS must be applied as recommendedby the developers.Relevant estimator of risk must be used : OR forCC designs and RR for SCCS designs	Report statistical method and estimator of risk used in the materials and methods section. Report specific estimator with confidence interval in the results section.Use adequate denomination for risk estimators in reports: OR and RR.
Sample size calculation	For CC, sample size calculation should be estimated based oncase-control method^22^. For SCCS, use the published samplesize formula to calculate the sample size for SCCS^16^.	Report the calculate sample size and all the elements necessary to reproduce the calculation
Sensitivity analyses	Sensitivity analyses must be conducted to check therobustness of particular methodology choices, particularlyrisk and control period selection choice.	Report all sensitivity analyses conducted and their results
Results reporting	N/A	Report the risk estimator and its 95% confidence interval.For CC, report count of discordant cases.For SCCS, report count of events in the different time periods.

## Discussion

We conducted a systematic review of case-only studies in pharmacoepidemiology published up to September 15, 2010, on MEDLINE, EMBASE and the SCCS method website [13]. We focused on CC and SCCS designs, the most popular case-only designs. Indeed, in the articles we screened, we only found 11 articles reporting a PSSA design and 8 using a screening method that fulfilled our eligibility criteria. In pharmacoepidemiology, case-only designs are predominantly used to assess safety. SCCS was originally developed to study adverse events of vaccines and is still mostly used for this purpose (76% of our articles). In contrast, CC has been used in a wider range of therapeutic areas.

These designs can still be considered to be emerging; their strengths and limitations are still being explored in particular settings [20], [21]. Accordingly, we found that numerous reports (42%) combined and compared case-only designs to more traditional ones, such as cohort and case-control designs. The most frequently reported rationale for using such designs was the elimination of bias due to time-invariant confounders between controls and cases. In general, case-only designs also seem worthwhile and attractive to investigators because they address the question of ‘why now?’, which may add information to traditional designs asking ‘why me ?’ [22].

Case-only designs were developed in a specific setting, implying specific validity assumptions adapted to these contexts. However, all these assumptions are not systematically fulfilled when the designs are used in different settings. In pharmacoepidemiology, we observed that all validity assumptions were fulfilled in 24% of CC and 40% of SCCS studies. For instance, in SCCS reports, events frequently affect the probability of further exposure. Applying these designs in inappropriate situations may lead to bias. Therefore at the time these studies are planned, these assumptions need to be checked and protocols to be adapted in order to fulfill them. When some validity assumptions are not fulfilled, the impact on the results needs to be discussed and conclusions should be drawn cautiously. Among reports including case-only studies compared to other design(s), we did not find that unfulfillment of all the assumptions was associated with discrepancy between case-only and conventional studies results in terms of statistical inference. However, the sample for this exploratory analysis was small and it is difficult to draw any firm conclusions from it with regard to the relevance of the proposed assumptions. Also, since case-only hypotheses do not address exactly the same questions as traditional ones [22], discrepant results among designs may not be directly attributed to a violation of validity assumptions. Comparing case-only results with another design’s results may still be relevant. While similar conclusions drawn from similar statistical results across designs would reinforce the conclusion on a particular question, discordant results may suggest at least the existence of biases within one (or more) of the performed designs. We considered that results were discordant when statistics were significant in one design and non significant in the other, or when the estimated association between exposure and event was in different directions, therefore leading to different conclusions across designs. Such discrepancy of inference among designs may merely reflect that case-only methods may control for specific biases (for instance fixed between-person confounders) and are sensitive to others (for instance measurement biases for drug intake), as opposed to conventional designs. It would be of interest to investigate further the impact of not fulfilling some assumptions, and also to extend the case-only designs to less strict validity assumptions would be useful to enhance the informativeness of case-only designs in pharmacoepidemiology. To overcome unavoidable ‘assumption issues’ alternative solutions have already been developed. For instance, for CC, in the case of a time-trend in exposure, the CTC design has been proposed [2]. Extensions of the SCCS design have been proposed as well, in the case of a probability of post-event exposure being affected by event occurrence, or in the case of event-dependent control period [23], [24]. When reading an article, it has to be possible to appreciate the robustness of the analysis; therefore, fulfillment of validity assumptions should be reported.

For the same reason, reporting relevant information in the results section is important. Solely reporting the risk estimate and its confidence interval does not provide adequate information to fully understand the specificity of a case-only analysis. However, adequate information will allow a comprehensive interpretation of the analysis, as well as the assessment of the internal validity of the study. In addition to the risk estimate, stating the count of subjects with discordant periods (exposed/unexposed) in CC studies, and the count of events in risk and control periods separately in SCCS studies, provides the reader with adequate information to appreciate how the analysis was performed. In half of the articles included, this information was partially missing or inadequate counts were provided, such as the count of exposed and non-exposed risk periods in CC design and the count of events, irrespective of risk/control periods in SCCS. This issue has recently been discussed by the team of the SCCS developers [25]. They stressed the importance of reporting the numbers of events in risk and control periods and of disclosing potential biases.

In case-only designs, specifying the risk period requires particular caution [26]: if the risk period is too long, too short or does not cover the true period at risk, the estimator may be biased toward the null [1], [9]. We found that 36% and 51% of CC and SCCS reports, respectively, adequately defined the risk period. In addition, sensitivity analyses are recommended to check the robustness of the risk period and control period choice, by modifying the onset, end or duration of the period. Only 17% of the reports described sensitivity analyses based on variation in the risk period, and 16% in the control period. In certain settings the event occurrence is only possible during specific periods at risk (eg pregnancy, hospitalization, driving, working). Therefore, time restrictions are required and, when appropriate, similar in risk and control periods. In our review, few reports described time restrictions for risk or control periods.

Regarding statistical issues, sample size calculation and power of case-only designs should be reported. In this review, only 11% of the articles reported a sample size calculation. For the CC design, the sample size issue was addressed and published by its developer in 2000 [27], stating that “Less than half as many subjects may be needed in a case-crossover study as in a traditional case – control study”. Sample size for SCCS was addressed and published, by the developer, in the context of surveillance of post vaccine adverse events, in 1996 [17], and a comprehensive sample size calculation formula was published in 2006 [18]. With regard to power considerations, two main comments may be drawn from our review. First, case-only designs often rely on data from pre-existing databases (administrative, insurance, hospital records and other healthcare-related databases) [28], which results in large sample sizes. However the actual cases count may remain low and insufficient. Secondly, authors often combined a case-only analysis with conventional one(s), as a secondary or exploratory analysis. The traditional design provided the basis for the sample size calculation, which was used for the exploratory case-only analysis, requiring a lower sample size. However, overall, we found that very few articles reported a sample size calculation, even when a large sample could not be expected from the use of an administrative database or when the case only design was not combined with another analysis. Also, reports without sample size calculation did not report an *a posteriori* power calculation either. Power considerations require caution in case-only analyses and it would be useful to clearly state the sample size and discuss its impact on the statistical analysis, even when using administrative databases or combining several designs. Ideally, an *a priori* sample size calculation is reported [18], [27]; if no calculation was performed, power considerations could be part of the discussion. Providing an *a posteriori* power calculation would help more readers to better interpret the results, particularly in the case of a non significant result. In pharmacoepidemiology, as adverse events are mostly very rare outcomes, information on power is key to ensuring accurate interpretation of results.

Although most reports described the use of proper statistical models and risk estimators, 31% of the articles were unclear as to the statistical model used. Two reports incorrectly presented their design as a case-crossover analysis: one compared cumulative drug doses between different time periods and the other was, in fact, a self-controlled case series analysis. Also, risk estimators’ terminology was not consistent, which might lead to misunderstanding regarding the analysis performed. Statistical analysis needs to be planned using the recommended models and risk estimators, implemented by the designs developers. More consistent risk estimator reporting would be welcome, preferably presenting odds ratios (OR) for CC and relative risk values (or incidence) for SCCS.

Our results highlight some issues to be improved on in planning and reporting case-only studies. These issues are: the applicability of the design to the context of the study, justification of risk and control periods (onset, time and duration), performing sensitivity analyses including those related to the risk and control period characteristics, reporting of sample size calculation and power discussion, statistical models and risk estimators, and reporting of adequate information regarding the analysis performed and results.

Our sample of reports may not be exhaustive. We focused on pharmacoepidemiology studies in human health and conducted our search in two of the main medical literature databases (MEDLINE and EMBASE) using keywords and few limits (English language); we also searched the references cited in selected (when appropriate) or included articles and on the SCCS method website [13]. As we mentioned above, three reviewers (SN, LB, FT) independently pre-tested our standardized form with a random set of 10 reports and every ambiguous data was discussed.

In conclusion, although the methodology of case-only designs is still being developed, these designs have been widely used in pharmacoepidemiology in the past decade. This review provides an overview of the use of both CC and SCCS in pharmacoepidemiology, covering various therapeutic areas. We found that pharmacoepidemiological studies did not always fulfill all of the validity assumptions for these designs. Authors should report this issue and the potential impact on results. Specificity of case-only design planning in pharmacoepidemiology remains an issue to be discussed and investigated thoroughly in the future [25], [27], [29]–[31]. We also found that results reporting often lacks key elements and impedes the assessment of the internal validity of the case-only analyses. Table 6 outlines the main points related to methodology issues to be addressed specifically in planning and reporting case-only studies. It is intended to be used in addition to the STROBE statement [14] for the specific field of case-only studies in pharmacoepidemiology. Our review highlights the need for improvement in reporting and planning in this context, and provides a first step in this direction.

## Supporting Information

Appendix S1 Case-only designs description and references
(DOC)

Appendix S2 Data collection form
(DOC)

Appendix S3 List of included articles
(DOC)
