# Supplementary material for: Case-Only Designs in Pharmacoepidemiology: A Systematic Review
Source: PLoS One. 2012 Nov 16;7(11):e49444. doi: 10.1371/journal.pone.0049444 (PMC3500300; doi:10.1371/journal.pone.0049444)
Supplement: Appendix S1 — Case-only designs description and references (DOC) [file pone.0049444.s001.doc]

**Appendix S1: Case-only designs description and references**

**Case-Crossover design [1]**

The CC design was introduced by Maclure in 1991 to study the short-term effects of intermittent exposures on the risk of acute outcomes. In a case-crossover study, case-subjects are their own self-matched controls by using pre-defined time period(s). Probability of exposure is compared between risk (or hazard) time-period(s) preceding the event of interest (where the exposure is considered associated to the event), and control period(s) defined in the observation period. Definition of these time periods (duration, etc.) depends on the studied outcome and exposure. The odds ratio for the outcome is estimated with a conditional logistic regression. Only discordant pairs, i.e. cases exposed only in the risk period or only in the control period, contribute to the analysis.

**Case-Time Control [2]**

Case-Time Control design adjusts a case-crossover analysis on a time-trend in the exposure estimated from a group of control subjects.

**Self-Controlled Case Series [3]**

Farrington proposed the self-controlled case-series (SCCS) design in 1995 to assess post-licensure adverse events related to vaccines, and more generally associations between acute outcome and transient exposure [4]. The SCCS design focuses on the relative incidence of an outcome between risk (post-exposure) and control time periods. Risk periods are defined during and/or after an exposure, when people are hypothesized to be at greater risk of the event, whereas control periods include all time period with baseline risk, which may occur both before and after the exposure. The statistics of this incidence rate ratio relies on a conditional Poisson regression.

Thus the CC design relates to a case control study (by comparing the probability of exposure between case and control periods), while the SCCS relates to a cohort study (by comparing the probability of events between exposed and non exposed periods).

**Prescription Sequence Analysis [5]**

To study the association between a drug and an adverse event, a prescription sequence analysis is based on the fact that occurrence of the adverse event is treated by prescription of a second drug. Prescription of the second drug in a defined time-period is used as a surrogate of the adverse event. Monte-Carlo simulation analysis is used to estimate the distribution of the prescription of the second drug within the define time period.

**Screening Method [6]**

Screening method was initially developed to assess the efficacy of vaccine. It estimates the protective effectiveness (PE) of a vaccine (or treatment) regarding the outcome of interest using the following equation: PE = 1 − [PCT/(1 − PCT)]/[PT/(1 − PT)],

where PCT is the proportion of cases treated and PT is the proportion of treated subjects (estimation of the population coverage for the treatment or vaccine)

**References**

[1] Maclure M (1991) The case-crossover design: a method for studying transient effects on the risk of acute events. Am J Epidemiol 133:144-53

[2] Suissa S (1995) The case-time-control design. Epidemiology 6:248-53

[3] Farrington CP (1995) Relative incidence estimation from case series for vaccine safety evaluation. Biometrics 51:228-35

[4] Whitaker HJ, Farrington CP, Spiessens B, Musonda P (2006) Tutorial in biostatistics: the self-controlled case series method. Stat Med 25:1768-97

[5] Petri H, de Vet HC, Naus J, Urquhart J (1988) Prescription sequence analysis: a new and fast method for assessing certain adverse reactions of prescription drugs in large populations. Stat Med 7:1171-5

[6] Farrington CP (1993) Estimation of vaccine effectiveness using the screening method. Int J Epidemiol 22:742-6.
