# Supplementary material for: Case-Only Designs in Pharmacoepidemiology: A Systematic Review
Source: PLoS One. 2012 Nov 16;7(11):e49444. doi: 10.1371/journal.pone.0049444 (PMC3500300; doi:10.1371/journal.pone.0049444)
Supplement: Appendix S2 — Data collection form (DOC) [file pone.0049444.s002.doc]

**Appendix S2: Data collection form**

DATA EXTRACTION FORM

Number |__|__|

Reader  SN  FT  LB

Title……………………………………………………………………………………………

…………………………………………………………………………………………………………………………………………………………………………………………………

First author……………………………………

Date of publication (year) |__|__|__|__|

**Journal**

Generalist Journal

Specialty Journal

Pharmacoepidemiology
 Others: ………………………….

GENERAL DATA

**Funding**

Public

Manufacturer

Both public and private

Not reported or unclear

No funding

Others:……………………………

Study aim

Safety  Yes  No

Effectiveness  Yes  No

Methodology  Yes  No

METHODS

STUDY DESIGN

Number of designs studying the same association used on the same database: |__|

Announced case only design:

Case-crossover (CC)  Yes  No

Case-time-control (CTC)  Yes  No

Self-controlled case series (SCCS)  Yes  No

##### Several designs are coupled Yes No

If yes, study coupled in a cohort study  Yes  No  Unclear

Study coupled in a case control study  Yes  No  Unclear

Several case only studies are used  Yes  No  Unclear

Others:……………………………….……………. Yes  No

Justification of the design choice

##### Justification of the design choice is reported Yes No

##### Acute event Yes No

Rare event  Yes  No

Recurrent exposure  Yes  No

Frequent exposure  Yes  No

Intermittent exposure  Yes  No

Short term risk  Yes  No

Suitable database (exposure history available and/or linkage possible and/or confounders not available on the database)  Yes  No

Not representative control group available  Yes  No

Risk of bias (confounding or selection bias) in conventional designs  Yes  No

Necessity of an easy, simple or rapid design  Yes  No

Others:…………………….………….…….. Yes  No

##### Part of the article where the choice of the design is justified

Title  Yes  No

Abstract  Yes  No

Introduction  Yes  No

Materials and methods  Yes  No

Discussion  Yes  No

DESIGN

Observation periods

Mean observation period: |__|__|__|__| months

The observation period is the same for each subject  Yes  No  Unclear

Pilot study

A previous pilot study is reported  Yes  No

SPECIFIC DESIGN

CASE-CROSSOVER AND CASE-TIME-CONTROL

Case window (current period if CTC) Characteristics

A case window is defined a priori, it is the period during which possible exposure to the risk factor of interest will be investigated in the previous history of cases.

***Case window definition***

The case window definition is based on physiological evidence  Yes  No  Unclear

The case window definition is based on practical issues  Yes  No  Unclear

The case window definition is sourced by a reference or validated by an expert group or based on previous study  Yes  No

##### Duration of the case window

The case window begins |__|__|__|__|__|  hours  days  weeks before the event

The case window ends |__|__|__|__|__|  hours  days  weeks before the event

Case window duration is the same for each subject  Yes  No  Unclear

*Case window selection*

##### The case window has restriction time Yes No Unclear

Control window (reference period if CTC) characteristics

Control window: Period used as a reference in the comparison

Every subject has the same number of control window  Yes  No

If yes, number of control windows per case window: |__|__|

##### Duration of the control window

Duration of a control window: |__|__|__|__|__|  hours  days  weeks

Every control window has the same duration  Yes  No

***Control windows selection (sensitivity analysis excluded)***

Immediately before the case window  Yes  No

One week or longer before the case window  Yes  No

Immediately after the case window  Yes  No

One week or longer after the case window  Yes  No

Before and after the case window  Yes  No

According to observation period  Yes  No

Randomized  Yes  No

Unclear  Yes  No

All the observation period but the case window  Yes  No

Control window with time restriction, before or after  Yes  No

Others:…………………………………………. Yes  No

SELF-CONTROLLED CASE-SERIES

Risk period Characteristics

Duration of the risk period: |__|__|__|__|__| days

The high-risk period begin |__|__|__|__|__| days after the exposure onset

The high risk period end |__|__|__|__|__| days after the exposure onset

The risk period is divided in several person-times  Yes  No  Unclear

Definition of the risk period

The risk period definition is based on physiological evidence  Yes  No  Unclear

The risk period definition is based on practical issues  Yes  No  Unclear

The risk period definition is based on previous or exploratory studies

Yes  No  Unclear

The risk period definition is validated by an experts group  Yes  No  Unclear

The risk period definition is sourced by a reference  Yes  No  Unclear

Risk period selection

Time restrictions of risk period

Time restriction of risk period  Yes  No  Unclear

Control period selection

The control period is all the observation period but the risk period  Yes  No  Unclear

The control period is a pre specified period  Yes  No  Unclear

If yes, number of control period |__|

Duration of control period |__|__|__|  Days  Weeks  Months

Others:…………………………………………………………… Yes  No  Unclear

VARIABLES

*EXPOSURE*

Number of studied exposures: |__|__|

Exposures:…………………………………………………………………………………………………………………………………………………………………………………………

**1. Type of exposure**

Hypnotics  Yes  No

Cardiovascular devices  Yes  No

Anti-inflammatory agents Yes  No

Vaccines  Yes  No

Gloves  Yes  No

Antidepressants  Yes  No

Condom  Yes  No

Anti-microbial agents  Yes  No

Asthma drugs  Yes  No Anticoagulants  Yes  No

Antihypertensive agents  Yes  No

Nicotine substitute  Yes  No

Diuretics  Yes  No

Antihyperlipidemic agents  Yes  No

Antiretroviral  Yes  No

Erectile dysfunction treatment…………

…………………………… Yes  No

Others:……………… Yes  No

**2. Characteristics of the exposures**

The exposure is rare………………………….……………… Yes  No  Don’t Know

The exposure is intermittent………………………………….. Yes  No  Don’t Know

The exposure induces short-term trigger…………………… Yes  No  Don’t Know

The exposure induces cumulative trigger…………………….. Yes  No  Don’t Know

The exposure is a short-term deterrent……………………….. Yes  No  Don’t Know

The exposure is a long-term deterrent……………….……….. Yes  No  Don’t Know

The exposure is non-adherence to the medication……………. Yes  No  Don’t Know

The exposure is changing medication (new dose or new drug)  Yes  No  Don’t Know

Other:…………………………………………………………  Yes  No

**3. Definition of the exposures**

The exposure definition (dosage, frequency and duration as appropriate) is clearly described ……………………………………………………... Yes  No

The exposures definition is sourced by a reference or validated by an expert group

Yes  No  Not Applicable

EVENTS

Number of studied events: |__|

Events:……………………………………………………………………………………………………………………………………………………………………………………………

**1. Type of events studied:**

Inflammatory disease  Yes  No

Infection  Yes  No

Hypersensitivity  Yes  No

Intussusceptions  Yes  No

Neurological symptoms  Yes  No

Road traffic accident  Yes  No

Cardiovascular disease  Yes  No

Gastrointestinal symptoms  Yes  No

Death  Yes  No

Fall  Yes  No

Psychiatric symptoms  Yes  No

Biological tests  Yes  No

Others:……………………………………………………………  Yes  No

**2. Characteristics of the events**

Rare event  Yes  No  Don’t Know

Acute event  Yes  No  Don’t Know

Sudden onset  Yes  No  Don’t Know

Irreversible event  Yes  No  Don’t Know

Recurrent event  Yes  No  Don’t Know

Others:………………………………………………..  Yes  No

**3. Events definition**

The event definition is clearly described  Yes  No

The event definition is sourced by a reference or validated by an expert group  Yes  No

**Events independence**

Multiple case windows were considered  Yes  No  Unclear  Not Applicable

If yes, the events independence is defined  Yes  No  Unclear

DATA SOURCES/MEASUREMENT

EXPOSURE

**1. Exposure data collection**

Case’s exposure data were extracted from:

Register  Yes  No

Clinical trial or cohort data)  Yes  No

Institution or hospital records  Yes  No

Reimbursement database  Yes  No

Primary care database  Yes  No

Self-report questionnaire or diary  Yes  No

Telephone call  Yes  No

Web site  Yes  No

Individual health book  Yes  No

Interview  Yes  No

Data available about exposure

Concomitant treatments  Yes  No  Unclear  Not Applicable

Others:…………………………... Yes  No

Mode of data collection

Data about exposure were collected prospectively  Yes  No  Unclear

**2. Measurement of the exposure**

The exposure is measured in the same way during case window and control window

Yes  No  Unclear  Not Applicable

EVENT

The protocol of the event’s collection is standardized  Yes  No  Unclear

**1. Event data collection**

Database where data about events were extracted

Register  Yes  No

Clinical trial or cohort data)  Yes  No

Institution or hospital records  Yes  No

Reimbursement database  Yes  No

Primary care database  Yes  No

Self-report questionnaire or diary  Yes  No

Telephone call  Yes  No

Web site  Yes  No

Individual health book  Yes  No

Interview  Yes  No

Data available about the events

Time of the event onset  Yes  No  Unclear

Co-morbidities  Yes  No  Unclear

Others:………………….. . Yes  No

Data collection mode

Data about event were collected prospectively

Yes  No  Unclear

STATISTICAL METHODS

**Study size**

Calculation of the sample size is reported  Yes  No

*If yes, sample size calculated: |__|__|__|__|__|*

**Statistical model**

Conditional logistic regression (CC) or Poisson regression (SCCS)  Yes  No

Semi parametric poisson regression model  Yes  No

Mantel-Haentzel  Yes  No

Others  Yes  No

Not reported or unclear  Yes  No

Not relevant  Yes  No

Confounding variables

Adjustment for confounding variables have been made  Yes  No  Unclear

If yes, the variables included in the model are described  Yes  No  Unclear

Interaction tests are reported  Yes  No

Sensitivity analysis

Sensitivity analysis are reported  Yes  No

If yes, the authors

Varied duration of case window  Yes  No

Varied duration of control window  Yes  No

Varied duration of induction period  Yes  No

Changed control window beginning  Yes  No

Changed control window number  Yes  No

Changed person-times duration  Yes  No

Changed person-times number  Yes  No

Changed exposure definition  Yes  No

Changed event definition  Yes  No

Others:……………………………..  Yes  No

Subgroups analysis

Subgroup analyses have been conducted  Yes  No

RESULTS

There is at least one significant result  Yes  No

If no, the authors discussed the problem of having a lack of power  Yes  No

OUTCOME DATA

A detailed dose-response gradient has been estimated  Yes  No  Not Applicable

Adjustment variables

Results presented are:

Adjusted  Yes  No  Unclear

Unadjusted  Yes  No  Unclear

*CASE-CROSSOVER/CASE-TIME-CONTROL*

The estimator of the risk is

Risk Ratio

Relative Risk

Odds Ratio

Relative Incidence

Incidence Rate

Excess Risk

IRR

Absolute risk

Others:…………….

*SELF-CONTROLLED CASE SERIES*

The estimator of the risk is

Risk Ratio

Relative Risk

Odds Ratio

Relative Incidence

Incidence Rate

Excess Risk

IRR

Absolute risk

Others:……………..

**CONCLUSION**

If different design on the same database to study the same association, the results obtained using different designs were concordant (both results were not significant or results were in the same direction)  Yes  No  Not Applicable  Partly

Methodological conclusions are reported  Yes  No

Efficient in standard application  Yes  No

Efficient in non standard application  Yes  No

Non efficient in standard application  Yes  No

Non efficient in non standard application  Yes  No

Requiring accurate definition and measurement of the outcome  Yes  No

Sensitive to bias (confounding, cases selection, indication bias)  Yes  No

Non-sensitive to bias (indication, time-unvarying bias)  Yes  No

Better than standard designs  Yes  No

Same as standard designs  Yes  No

Inferior as standard designs  Yes  No

Others…………………………………………………………… Yes  No

**DESIGN ASSUMPTIONS**

**CC**

Acute event  Yes  No  Unclear

Rare event  Yes  No  Unclear

Intermittent exposure  Yes  No  Unclear

Exchangeability of the probability of exposure  Yes  No  Unclear

Opportunity of event  Yes  No  Unclear

**SCCS**

Rare or recurrent event Yes  No  Unclear

Event that does not affect the probability of future exposure  Yes  No  Unclear

If the event is recurrent, the independence between two consecutive events is defined

Yes  No  Unclear

Transient/intermittent event Yes  No  Unclear

The collection of exposures and events were independent  Yes  No  Unclear

Event did not affect the short term mortality probability meaning that the observation period is independent of events Yes  No  Unclear
