# Supplementary material for: Case-Only Designs in Pharmacoepidemiology: A Systematic Review
Source: PLoS One. 2012 Nov 16;7(11):e49444. doi: 10.1371/journal.pone.0049444 (PMC3500300; doi:10.1371/journal.pone.0049444)
Supplement: Appendix S3 — List of included articles (DOC) [file pone.0049444.s003.doc]

**Appendix S3: List of included articles**

1. Aberra FN, Brensinger CM, Bilker WB, Lichtenstein GR, Lewis JD (2005) Antibiotic use and the risk of flare of inflammatory bowel disease. Clin Gastroenterol Hepatol 3(5):459-65
2. Ali M, Canh GD, Clemens JD, Park JK, von Seidlein L et al. (2005) The use of a computerized database to monitor vaccine safety in Viet Nam. Bull World Health Organ 83(8):604-10
3. Andrews N, Miller E, Waight P, Farrington P, Crowcroft N et al. (2001) Does oral polio vaccine cause intussusceptions in infants? Evidence from a sequence of three self-controlled cases series studies in the United Kingdom. Eur J Epidemiol 17(8):701-6
4. Andrews N, Stowe J, Miller E, Taylor B (2007) Post-licensure safety of the meningococcal group C conjugate vaccine. Hum Vaccin 3(2):59-63.
5. Balkrishnan R, Christensen DB (2000). Inhaled corticosteroid nonadherence and immediate avoidable medical events in older adults with chronic pulmonary ailments. J Asthma 37(6):511-7
6. Barbone F, McMahon AD, Davey PG, Morris AD, Reid IC et al.(1998) Association of road-traffic accidents with benzodiazepine use. Lancet 24;352(9137):1331-6.
7. Biskupiak JE, Brixner DI, Howard K, Oderda GM (2006) Gastrointestinal complications of over-the-counter nonsteroidal antiinflammatory drugs. J Pain Palliat Care Pharmacother 20(3):7-14.
8. Cameron JC, Walsh D, Finlayson AR, Boyd JH (2006) Oral polio vaccine and intussusceptions: A Data Linkage Study using Records for Vaccination and Hospitalization. Am J Epidemiol 163:528–33
9. Cohen AD, Bonneh DY, Reuveni H, Vardy DA, Naggan L et al. (2005) Drug exposure and psoriasis vulgaris: case-control and case-crossover studies. Acta Derm Venereol 85(4):299-303.
10. Confavreux C, Suissa S, Saddier P, Bourdès V, Vukusic S (2001) Vaccines in Multiple Sclerosis Study Group. Vaccinations and the risk of relapse in multiple sclerosis. Vaccines in Multiple Sclerosis Study Group. N Engl J Med 344(5):319-26.
11. Corrao G, Zambon A, Faini S, Bagnardi V, Leoni O et al. (2005) Short-acting inhaled beta-2-agonists increased the mortality from chronic obstructive pulmonary disease in observational designs. J Clin Epidemiol 58(1):92-7.
12. Dourado I, Cunha S, Teixeira MG, Farrington CP, Melo A et al. (2000) Outbreak of aseptic meningitis associated with mass vaccination with a urabe-containing measles-mumps-rubella vaccine: implications for immunization programs. Am J Epidemiol 151(5):524-30.
13. Etienney I, Beaugerie L, Viboud C, Flahault A (2003) Non-steroidal anti-inflammatory drugs as a risk factor for acute diarrhoea: a case crossover study. Gut 52(2):260-3.
14. Fagot JP, Mockenhaupt M, Bouwes-Bavinck JN, Naldi L, Viboud C et al. (2001) Nevirapine and the risk of Stevens-Johnson syndrome or toxic epidermal necrolysis. AIDS 15(14):1843-8.
15. Farrington P, Pugh S, Colville A, Flower A, Nash J et al. (1995) A new method for active surveillance of adverse events from diphtheria/tetanus/pertussis and measles/mumps/rubella vaccines. Lancet 345(8949):567-9.
16. Farrington CP, Miller E, Taylor B (2001) MMR and autism: further evidence against a causal association. Vaccine 19(27):3632-5.
17. Finkelstein Y, Schechter T, Garcia-Bournissen F, Kirby M, Nurmohamed L et al. (2007) Is morphine exposure associated with acute chest syndrome in children with vaso-occlusive crisis of sickle cell disease? A 6-year case-crossover study. Clin Ther 29(12):2738-43
18. Gislason GH, Jacobsen S, Rasmussen JN, Rasmussen S, Buch P et al. (2006) Risk of death or reinfarction associated with the use of selective cyclooxygenase-2 inhibitors and nonselective nonsteroidal antiinflammatory drugs after acute myocardial infarction. Circulation 113(25):2906-13.
19. Hambidge SJ, Glanz JM, France EK, Maclure D, Xu S et al. (2006) Safety of trivalent inactivated influenza vaccine in children 6 to 23 months old. JAMA 296(16):1990-7.
20. Handoko KB, Zwart-van Rijkom JE, Hermens WA, Souverein PC, Egberts TC (2007) Changes in medication associated with epilepsy-related hospitalisation: a case-crossover study. Pharmacoepidemiol Drug Saf 16(2):189-96.
21. Hauser T, Mahr A, Metzler C, Coste J, Sommerstein R et al. (2008) The leucotriene receptor antagonist montelukast and the risk of Churg-Strauss syndrome: a case-crossover study. Thorax 63(8):677-82.
22. Hebert C, Delaney JA, Hemmelgarn B, Lévesque LE, Suissa S (2007) Benzodiazepines and elderly drivers: a comparison of pharmacoepidemiological study designs. Pharmacoepidemiol Drug Saf 16(8):845-9
23. Hernández-Díaz S, Hernán MA, Meyer K, Werler MM, Mitchell AA (2003) Case-crossover and case-time-control designs in birth defects epidemiology. Am J Epidemiol 158(4):385-91
24. Hocine MN, Farrington CP, Touzé E, Whitaker HJ, Fourrier A at al. (2007) Hepatitis B vaccination and first central nervous system demyelinating events: reanalysis of a case-control study using the self-controlled case series method. Vaccine 25(31):5938-43
25. Hubbard R, Farrington P, Smith C, Smeeth L, Tattersfield A (2003) Exposure to tricyclic and selective serotonin reuptake inhibitor antidepressants and the risk of hip fracture. Am J Epidemiol 158(1):77-84.
26. Hubbard R, Lewis S, Smith C, Godfrey C, Smeeth L et al. (2005) Use of nicotine replacement therapy and the risk of acute myocardial infarction, stroke, and death. Tob Control 14(6):416-21.
27. Hubbard R, Lewis S, West J, Smith C, Godfrey C et al. (2005) Bupropion and the risk of sudden death: a self-controlled case-series analysis using The Health Improvement Network. Thorax 60(10):848-50.
28. Hughes RA, Charlton J, Latinovic R, Gulliford MC (2006) No association between immunization and Guillain-Barré syndrome in the United Kingdom, 1992 to 2000. Arch Intern Med 166(12):1301-4.
29. Hunter DJ, York M, Chaisson CE, Woods R, Niu J et al. (2006) Recent diuretic use and the risk of recurrent gout attacks: the online case-crossover gout study. J Rheumatol 33(7):1341-5. Erratum in: J Rheumatol 2006 ;33(8):1714.
30. Hutchinson KB, Kip KE, Ness RB. Condom use and its association with bacterial vaginosis and bacterial vaginosis-associated vaginal microflora. Epidemiology.2007;18(6):702-8
31. Juurlink DN, Stukel TA, Kwong J, Kopp A, McGeer A et al. (2006) Guillain-Barré syndrome after influenza vaccination in adults: a population-based study. Arch Intern Med 166(20):2217-21.
32. Ki M, Park T, Yi SG, Oh JK, Choi B (2003) Risk analysis of aseptic meningitis after measles-mumps-rubella vaccination in Korean children by using a case-crossover design. Am J Epidemiol 157(2):158-65.
33. Kjaer D, Horvath-Puhó E, Christensen J, Vestergaard M, Czeizel AE et al. (2007) Use of phenytoin, phenobarbital, or diazepam during pregnancy and risk of congenital abnormalities: a case-time-control study. Pharmacoepidemiol Drug Saf 16(2):181-8.
34. Kramarz P, Destefano F, Gargiullo PM, Chen RT, Lieu TA et al. (2001) Does influenza vaccination prevent asthma exacerbations in children? J Pediatr 138(3):306-10.
35. Miller E, Waight P, Farrington CP, Andrews N, Stowe J et al. (2001) Idiopathic thrombocytopenic purpura and MMR vaccine. Arch Dis Child 84(3):227-9.
36. Miller E, Andrews N, Waight P, Taylor B (2003) Bacterial infections, immune overload, and MMR vaccine. Measles, mumps, and rubella. Arch Dis Child 88(3):222-3.
37. Miller E, Andrews N, Grant A, Stowe J, Taylor B (2005) No evidence of an association between MMR vaccine and gait disturbance. Arch Dis Child 90(3):292-6.
38. Miller E, Andrews N, Stowe J, Grant A, Waight P et al. (2007) Risks of convulsion and aseptic meningitis following measles-mumps-rubella vaccination in the United Kingdom. Am J Epidemiol 165(6):704-9.
39. Mittleman MA, Maclure M, Glasser DB (2005) Evaluation of acute risk for myocardial infarction in men treated with sildenafil citrate. Am J Cardiol. 96(3):443-6.
40. Mullooly JP, Crane B, Chun C (2006) Trivalent inactivated influenza vaccine safety in children: assessing the contribution of telephone encounters. Vaccine 24(13):2256-63.
41. Murphy TV, Gargiullo PM, Massoudi MS, Nelson DB, Jumaan AO et al. (2001) Intussusception among infants given an oral rotavirus vaccine. N Engl J Med 344(8):564-72. Erratum in: N Engl J Med 2001;344(20):1564. Livingood, JR [corrected to Livengood, JR].
42. Mutsch M, Zhou W, Rhodes P, Bopp M, Chen RT et al. (2004) Use of the inactivated intranasal influenza vaccine and the risk of Bell's palsy in Switzerland. N Engl J Med 350(9):896-903.
43. Shuto H, Imakyure O, Matsumoto J, Egawa T, Jiang Y et al. (2010) Medication use as a risk factor for inpatient falls in an acute care hospital: a case-crossover study. Br J Clin Pharmacol 69(5):535-42.
44. Payne DC, Aranas A, McNeil MM, Duderstadt S, Rose CE Jr (2007) Concurrent vaccinations and U.S. military hospitalizations. Ann Epidemiol 17(9):697-703.
45. Sardiñas MA, Cárdenas AZ, Marie GC, Peña MS, Santiago MA et al. (2001) Lack of association between intussusception and oral polio vaccine in Cuban children. Eur J Epidemiol 17(8):783-7.
46. Smeeth L, Thomas SL, Hall AJ, Hubbard R, Farrington P et al. (2004) Risk of myocardial infarction and stroke after acute infection or vaccination. N Engl J Med 351(25):2611-8.
47. Sorock GS, Lombardi DA, Peng DK, Hauser R, Eisen EA et al . (2004) Glove use and the relative risk of acute hand injury: a case-crossover study. J Occup Environ Hyg 1(3):182-90.
48. Stowe J, Andrews N, Wise L, Miller E (2006) Bell's palsy and parenteral inactivated influenza vaccine. Hum Vaccin 2(3):110-2.
49. Stowe J, Kafatos G, Andrews N, Miller E (2008) Idiopathic thrombocytopenic purpura and the second dose of MMR. Arch Dis Child 93(2):182-3.
50. Sturkenboom MC, Middelbeek A, de Jong van den Berg LT, van den Berg PB, Stricker BH et al. (1995) Wesseling H. Vulvo-vaginal candidiasis associated with acitretin. J Clin Epidemiol 48(8):991-7.
51. Suissa S (1995) The case-time-control design. Epidemiology 6(3):248-53.
52. Tata LJ, Fortun PJ, Hubbard RB, Smeeth L, Hawkey CJ et al. (2005) Does concurrent prescription of selective serotonin reuptake inhibitors and non-steroidal anti-inflammatory drugs substantially increase the risk of upper gastrointestinal bleeding? Aliment Pharmacol Ther 22(3):175-81.
53. Tata LJ, West J, Harrison T, Farrington P, Smith C et al. (2003) Does influenza vaccination increase consultations, corticosteroid prescriptions, or exacerbations in subjects with asthma or chronic obstructive pulmonary disease? Thorax 58(10):835-9
54. Tata LJ, West J, Smith C, Farrington P, Card T et al. (2005) General population based study of the impact of tricyclic and selective serotonin reuptake inhibitor antidepressants on the risk of acute myocardial infarction. Heart 91(4):465-71
55. Taylor B, Andrews N, Stowe J, Hamidi-Manesh L, Miller E (2007) No increased risk of relapse after meningococcal C conjugate vaccine in nephrotic syndrome. Arch Dis Child 92(10):887-9
56. DeStefano F, Chen RT (2000) Autism and measles, mumps, and rubella vaccine: No epidemiological evidence for a causal association. J Pediatr 36(1):125-6
57. Wang PS, Schneeweiss S, Glynn RJ, Mogun H, Avorn J (2004) Use of the case-crossover design to study prolonged drug exposures and insidious outcomes. Ann Epidemiol 14(4):296-303
58. Ward KN, Bryant NJ, Andrews NJ, Bowley JS, Ohrling A et al. (2007) Risk of serious neurologic disease after immunization of young children in Britain and Ireland. Pediatrics 120(2):314-21
59. Warner L, Macaluso M, Austin HD, Kleinbaum DK, Artz L et al. (2005) Application of the case-crossover design to reduce unmeasured confounding in studies of condom effectiveness. Am J Epidemiol 161(8):765-73
60. Azoulay L, Blais L, Koren G, LeLorier J, Bérard A (2008) Isotretinoin and the risk of depression in patients with acne vulgaris: a case-crossover study. J Clin Psychiatry 69(4):526-32
61. Delaney JA, Suissa S (2009) The case-crossover study design in pharmacoepidemiology. Stat Methods Med Res 18(1):53-65. Epub 2008 Sep 2.
62. Douglas IJ, Smeeth L (2008) Exposure to antipsychotics and risk of stroke: self controlled case series study. BMJ 337:a1227.
63. Durán-Barragán S, McGwin G Jr, Vilá LM, Reveille JD, Alarcón GS (2008) LUMINA (LIX): a multiethnic US cohort. Angiotensin-converting enzyme inhibitors delay the occurrence of renal involvement and are associated with a decreased risk of disease activity in patients with systemic lupus erythematosus--results from LUMINA (LIX): multiethnic US cohort. Rheumatology 47(7):1093-6
64. Gibson JE, Hubbard RB, Smith CJ, Tata LJ, Britton JR et al. (2009) Use of self-controlled analytical techniques to assess the association between use of prescription medications and the risk of motor vehicle crashes. Am J Epidemiol 169(6):761-8
65. Gislason GH, Rasmussen JN, Abildstrom SZ, Schramm TK, Hansen ML et al. (2009) Increased mortality and cardiovascular morbidity associated with use of nonsteroidal anti-inflammatory drugs in chronic heart failure. Arch Intern Med 169(2):141-9.
66. Grosso A, Douglas I, Hingorani A, MacAllister R, Smeeth L (2008) Post-marketing assessment of the safety of strontium ranelate; a novel case-only approach to the early detection of adverse drug reactions. Br J Clin Pharmacol 66(5):689-94.
67. Grosso A, Douglas I, Hingorani A, MacAllister R, Smeeth L (2009) Oral bisphosphonates and risk of atrial fibrillation and flutter in women: a self-controlled case-series safety analysis. PLoS One 4(3):e4720. Epub 2009 Mar 6.
68. Hallas J, Bjerrum L, Støvring H, Andersen M (2008) Use of a prescribed ephedrine/caffeine combination and the risk of serious cardiovascular events: a registry-based case- crossover study. Am J Epidemiol 168(8):966-73.
69. Molokhia M, McKeigue P, Curcin V, Majeed A (2008) Statin induced myopathy and myalgia: time trend analysis and comparison of risk associated with statin class from 1991-2006. PLoS One 3(6):e2522.
70. Schelleman H, Bilker WB, Brensinger CM, Han X, Kimmel SE et al. (2008) Warfarin with fluoroquinolones, sulfonamides, or azole antifungals: interactions and the risk of hospitalization for gastrointestinal bleeding. Clin Pharmacol Ther 84(5):581-8. Epub 2008 Aug 6.
71. Stowe J, Andrews N, Wise L, Miller E (2009) Investigation of the temporal association of Guillain-Barre syndrome with influenza vaccine and influenzalike illness using the United Kingdom General Practice Research Database. Am J Epidemiol 169(3):382-8. Epub 2008 Nov 24
72. Stowe J, Andrews N, Taylor B, Miller E (2009) No evidence of an increase of bacterial and viral infections following Measles, Mumps and Rubella vaccine. Vaccine 27(9):1422-5. Epub 2009 Jan 13.
73. Zambon A, Polo Friz H, Contiero P, Corrao G (2009) Effect of macrolide and fluoroquinolone antibacterials on the risk of ventricular arrhythmia and cardiac arrest: an observational study in Italy using case-control, case-crossover and case-time-control designs. Drug Saf 32(2):159-67
74. Grosso A, Douglas I, Hingorani AD, MacAllister R, Hubbard R et al. (2009) Inhaled tiotropium bromide and risk of stroke. Br J Clin Pharmacol 68(5):731-6.
75. Park-Wyllie LY, Mamdani MM, Li P, Gill SS, Laupacis A et al. (2009) Cholinesterase inhibitors and hospitalization for bradycardia: a population-based study. PLoS Med 6(9):e1000157. Epub 2009 Sep 29
76. Rifkin SB, Smith MR, Brotman RM, Gindi RM, Erbelding EJ (2009) Hormonal contraception and risk of bacterial vaginosis diagnosis in an observational study of women attending STD clinics in Baltimore, MD. Contraception 80(1):63-7. Epub 2009 Mar 4
77. Dall M, Schaffalitzky de Muckadell OB, Lassen AT, Hansen JM et al. (2009) An association between selective serotonin reuptake inhibitor use and serious upper gastrointestinal bleeding. Clin Gastroenterol Hepatol 7(12):1314-21. Epub 2009 Aug 26
78. Naleway AL, Belongia EA, Donahue JG, Kieke BA, Glanz JM (2009) Risk of immune hemolytic anemia in children following immunization. Vaccine 27(52):7394-7. Epub 2009 Sep 18
79. Douglas IJ, Evans SJ, Pocock S, Smeeth L (2009) The risk of fractures associated with thiazolidinediones: a self-controlled case-series study. PLoS Med 6(9):e1000154. Epub 2009 Sep 29
80. Greene SK, Kulldorff M, Lewis EM, Li R, Yin R et al. (2010) Near real- time surveillance for influenza vaccine safety: proof-of-concept in the Vaccine Safety Datalink Project. Am J Epidemiol 171(2):177-88.
81. Velthove KJ, Leufkens HG, Schweizer RC, van Solinge WW, Souverein PC (2010) Medication changes prior to hospitalization for obstructive lung disease: a case-crossover study. Ann Pharmacother 44(2):267-73.
82. Olesen JB, Hansen PR, Erdal J, Abildstrøm SZ, Weeke P et al. (2010) Antiepileptic drugs and risk of suicide: a nationwide study. Pharmacoepidemiol Drug Saf 19(5):518-24
83. Gold M, Dugdale S, Woodman RJ, McCaul KA (2010) Use of the Australian Childhood Immunisation Register for vaccine safety data linkage. Vaccine 28(26):4308-11.
84. Grimaldi-Bensouda L, Abenhaim L, Michaud L, Mouterde O, Jonville-Béra AP et al. (2010) Clinical features and risk factors for upper gastrointestinal bleeding in children: a case-crossover study. Eur J Clin Pharmacol 66(8):831-7.
85. Fosbøl EL, Folke F, Jacobsen S, Rasmussen JN, Sørensen R et al. (2010) Cause-specific cardiovascular risk associated with nonsteroidal antiinflammatory drugs among healthy individuals. Circ Cardiovasc Qual Outcomes 3(4):395-405.
86. Shuto H, Imakyure O, Matsumoto J, Egawa T, Jiang Y et al. (2010) Medication use as a risk factor for inpatient falls in an acute care hospital: a case-crossover study. Br J Clin Pharmacol 69(5):535-42.
87. Lee CH, Wang JD, Chen PC (2010) Increased risk of hospitalization for acute hepatitis in patients with previous exposure to NSAIDs. Pharmacoepidemiol Drug Saf 19(7):708-14.
88. Schelleman H, Bilker WB, Brensinger CM, Wan F, Hennessy S (2010) Anti-infectives and the risk of severe hypoglycemia in users of glipizide or glyburide. Clin Pharmacol Ther 88(2):214-22.
89. Luo X, Sorock GS (2008) Analysis of recurrent event data under the case-crossover design with applications to elderly falls. Stat Med 27(15):2890-901
90. Viboud C, Boëlle PY, Kelly J, Auquier A, Schlingmann J et al. (2001) Comparison of the statistical efficiency of case-crossover and case-control designs: application to severe cutaneous adverse reactions. J Clin Epidemiol 54(12):1218-27.
91. Chang CH, Shau WY, Kuo CW, Chen ST, Lai MS (2010) Increased risk of stroke associated with nonsteroidal anti-inflammatory drugs: a nationwide case-crossover study. Stroke 41(9):1884-90. Epub 2010 Jul 29
92. Gagne JJ, Avorn J, Shrank WH, Schneeweiss S (2010) Refilling and switching of antiepileptic drugs and seizure-related events. Clin Pharmacol Ther 88(3):347-53.
93. Huang WT, Gargiullo PM, Broder KR, Weintraub ES, Iskander JK et al. (2010) Lack of association between acellular pertussis vaccine and seizures in early childhood. Pediatrics 126(2):263-9.
